# Method of micro‐sampling human dentine collagen for stable isotope analysis

**DOI:** 10.1002/rcm.9305

**Published:** 2022-04-28

**Authors:** Mandi J. Curtis, Julia Beaumont, Fadil Elamin, Andrew S. Wilson, Hannah E. C. Koon

**Affiliations:** ^1^ School of Archaeological and Forensic Sciences University of Bradford Bradford UK; ^2^ Institute of Dentistry, Bart's and The London School of Medicine and Dentistry Queen Mary University of London London UK; ^3^ Khartoum Centre for Research and Medical Training Khartoum Sudan

## Abstract

**Rationale:**

Sampling of dentine for stable carbon (δ^13^C) and nitrogen (δ^15^N) isotope ratios in the direction of tooth growth allows the study of temporal changes to the diet and physiological stress of an individual during tooth formation. Current methods of sampling permanent teeth using 1 mm increments provide temporal resolution of 6–9 months at best depending on the tooth chosen. Although this gives sufficient sample sizes for reliable analysis by mass spectrometry, sectioning the dentine across the incremental structures results in a rolling average of the isotope ratios. A novel method of incremental dentine collagen sampling has been developed to decrease the collagen increment size to 0.35 mm along the incremental structures, thus reducing averaging and improving the temporal resolution of short‐term changes within the δ^13^C and δ^15^N values.

**Methods:**

This study presents data for a MicroMill‐assisted sampling method that allows for sampling at 0.35 mm width × 1 mm depth increments following the incremental growth pattern of dentine. A NewWave MicroMill was used to sample the demineralised dentine section of modern donated human third molars from Sudan and compared to data from the same teeth using the 1 mm incremental sectioning method 2 established by Beaumont et al.

**Results:**

The δ^13^C and δ^15^N isotopic data showed an increased temporal resolution, with each increment providing data for 2–4 months of dentine formation.

**Conclusions:**

The data show the potential of this method for studying dietary reconstruction, nutritional stress, and physiological change with greater temporal resolution potentially to seasonal level and with less attenuation of the δ^13^C and δ^15^N values than was previously possible from human dentine.

## 1 INTRODUCTION

Dietary reconstruction using stable carbon and nitrogen isotope ratios (δ^13^C and δ^15^N) from bulk sampling of collagen from both bone and dentine has become a useful tool for understanding the lifeways of past individuals. Bulk dentine provides data from childhood and adolescence averaged over the period of tooth formation, whereas bulk bone gives data which has been averaged over longer periods of life. In adults, differing rates of bone turnover allow us to investigate data over 10 years or more, and in rib bones, from the last 5 years of life.^1^, ^2^, ^3^, ^4^, ^5^, ^6^, ^7^ However, the use of incremental dentine sampling has allowed us to study dietary shifts related to events such as breastfeeding, weaning, and physiological changes caused by undernutrition during tooth formation previously invisible in a single bulk sample.^8^, ^9^, ^10^ Dentine is an ideal material for incremental sampling research because it forms in predictable temporal increments and does not remodel after formation.^11^ δ^13^C and δ^15^N variations in the dentine collagen incremental profiles show clear patterns that have allowed researchers to distinguish between dietary and physiological changes.^12^, ^13^, ^14^, ^15^, ^16^, ^17^, ^18^ Different studies have used variations on the early sampling methods to improve temporal resolution and reduce averaging with varying success.^4^, ^5^, ^9^, ^14^, ^19^, ^20^

Teeth are often well preserved in archaeological sites,^21^ and in the absence of other incrementally forming tissues such as hair and nails, the development of reliable sampling methods has made teeth a preferred resource for research on dietary changes and life events in childhood and adolescence.^22^, ^23^, ^24^, ^25^

Methods of incremental sampling were previously limited by the amount of collagen required to obtain reliable data using a continuous‐flow isotope ratio mass spectrometer (IRMS). Fuller et al (2003) used deciduous and permanent teeth divided samples into 3–4 horizontal sections to study the breastfeeding and weaning patterns of archaeological remains from Wharram Percy, England.^20^ Eerkens et al (2011) used permanent first molars divided into 5–10 horizontal incremental sections to study weaning and childhood diets of six individuals from Marsh Creek banks in central California.^4^ Burt and Garvie‐Lok (2013) developed a micro‐sampling method on modern deciduous teeth from Canadian individuals to study pre‐ and post‐neonatal line sections to observe different life stages.^19^ The method involved punching disks out of the dentine on both sides of the neonatal line. Beaumont et al (2013) developed methods for sampling dentine collagen in 1 mm increments which excluded the filtration stage and thus increased the yield for each section.^9^, ^26^ For Beaumont Method 2 the whole root of a multi‐rooted tooth, or half of a single rooted tooth from crown to apex, is demineralised under refrigeration in 0.5 M HCl, and then sectioned by hand using a metal ruler and scalpel. The 1 mm increments are then denatured by heating at 70°C in a pH 3 solution of de‐ionised water and HCl, then frozen and freeze‐dried.^9^ This results in sufficient collagen for duplicate 0.5 mg samples to be weighed into tin capsules and measured using IRMS. This method was used on 19th‐century teeth from London to investigate childhood diet and migration to London for survivors of the Great Irish famine.^22^

**FIGURE 1:**
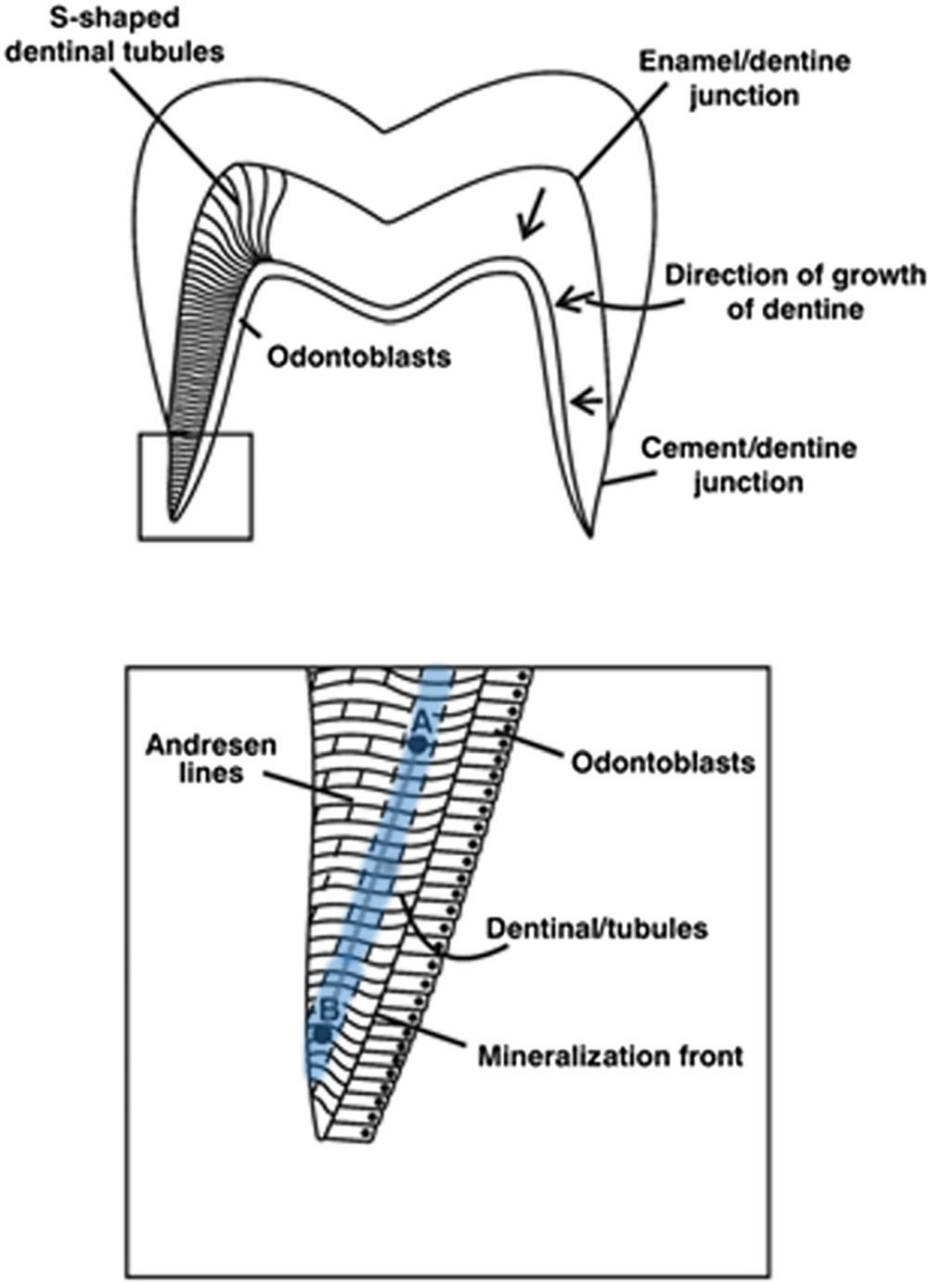
A diagram showing the direction of dentine development in the root of a human molar tooth, the relationship between Andresen bands and the mineralising front, and points A and B within the same Andresen band. Adapted from Beaumont et al, 2013^9^ [Color figure can be viewed at wileyonlinelibrary.com]

Root dentine is formed in a pattern similar to a series of concentric cones with varying angles of formation within different sections of the tooth. For example, in the crown of a molar, the dentine growth is nearly horizontal while the angle increases as tooth growth proceeds down the root relative to the pulp cavity and also changes as it approaches the root apex.^4^ Henderson et al (2014) adjusted Beaumont et al (2013) Method 2 by sectioning the demineralised dentine with five smaller sections in the crown and five larger sections from the remaining root in an attempt to make the time represented in each increment equal.^5^ With the exception of Burt and Garvie‐Lok (2013) all incremental methods involved cutting sections transversely from the crown to the apex at predetermined intervals resulting in analysis of multiple formation periods.^27^ Though proven to be effective in providing temporal resolution which is greatly improved over bulk sampling, there is still an averaging of the isotopic values because of the overlapping growth patterns of the developmental structures (Andresen bands). Andresen bands are patterns formed by diurnal rhythms of dentine formation causing different densities that can be seen under light microscope in both intact and demineralised dentine (Figure 1).^28^ Sampling in the direction of these bands rather than transversely across them would result in reducing the averaging of the isotopic signal between formation ages; however, this is a difficult task because they are visible only under a microscope.^9^, ^29^

Method development has been undertaken in Czermak et al (2018), Czermak et al (2020), and Lee et al (2020) to address issues caused by averaging of isotopic ratios that occurred when sectioning transversely through the demineralised dentine collagen.^30^, ^31^, ^32^ Czermak et al (2018) used high‐resolution light microscopy images to assist in directionality of incremental growth structures to increase the temporal resolution. The method cut microchunks in 1 mm increments along the growth lines of the dentine collagen, and 0.5 mm incremental microchunks were taken from the dentine collagen in the crown. Czermak et al (2020) further attempted to limit the averaging of the isotopic ratios within incremental sampling by eliminating the potential of secondary and tertiary dentine and cementum from contaminating the primary dentine collagen samples. This was done by implementing the use of a 1 mm diameter biopsy punch to remove increments from demineralised dentine collagen. Our aim in this study was to develop a micro‐sampling method that can be reliably used for sampling dentine collagen and will both improve the temporal resolution and reduce the averaging of δ^13^C and δ^15^N. The method we describe here uses a NewWave MicroMill™ to add a level of precision to the incremental sampling process that is not guaranteed when sampling by hand. Though similar in concept to previous sampling methods, this novel method reduces the amount of sample lost while significantly increasing the temporal resolution of the isotope data.

## 2 MATERIALS AND METHODS

### 2.1 Materials

Three modern third molars (M3) from adult females were analysed for this study (ET‐7889, ET‐9644, and ET‐7891). The teeth were collected as part of routine dental procedures at the Khartoum Centre for Research and Medical Training in Khartoum, Sudan, in 2011 for research purposes, and are part of a group collected to investigate changes in the appearance of microscopic dentine structures where teeth are formed during parturition (including the tooth described by Dean and Elamin in 2019^33^).

The individuals who provided samples signed consent forms to approve the use of their tissue for research, and the project was approved by the authorities at the University Hospital, Khartoum: the samples have been anonymised and personal data were processed by the Ethical Tissue Bank at the University of Bradford, UK.^33^ Modern teeth were used for the development of this method, in part, to eliminate the potential issues associated with collagen preservation within archaeological samples.

The samples have been previously analysed, using Method 2 by Beaumont et al (2013)^26^ by Jackson (2017).^34^ Data from the previous analysis would be used as a control for this method. The developmental ages were determined using the London Dental Atlas.^35^ M3 development occurs between the ages 8 and 23 years.

### 2.2 Standards, chemicals, and instruments

Chemicals used for this research were U.V. Glue (Bondic®, Cambridge, UK), and hydrochloric acid diluted to 0.5 M using de‐ionised H_2_O. The instruments used for sectioning dentine samples were Leica SP1600 Saw Microtome and ESI New Wave Micromill. The instrument used for analysing collagen samples was an EA IsoLink™ IRMS System, using Thermo Delta V Advantage IRMS coupled to a Thermo Flash 1112 Elemental Analyser via a ConFlo III.

Results are reported using delta (δ) notation in parts per thousand (per mil or ‰) relative to international standards. The carbon isotope ratios are expressed relative to Vienna Pee Dee Belemnite (VPDB), and the nitrogen isotope ratios are expressed relative to AIR (AIR N_2_).^36^, ^37^ The analytical error is 0.2‰. International Atomic Energy Agency (IAEA) standards 600, N1, CH3 and laboratory in‐house standards fish gel and bovine liver were used when analysing the collagen samples. δ^13^C and δ^15^N values for all standards used are listed in Table 1. Standards were distributed throughout and analysed with the samples in each analytical run.

**TABLE 1:** International and University of Bradford Stable Light Isotope Laboratory in‐house standards for IRMS analysis

	Analyte	Value	Unit	SD
IAEA‐600, caffeine	δ^13^C	−27.771	‰_VPDB_	0.043
	δ^15^N	1	‰_air N2_	0.2
IAEA‐N‐1, ammonium sulphate	δ^15^N	0.4	‰_air N2_	0.2
IAEA‐CH‐3, cellulose	δ^13^C	−24.724	‰_VPDB_	0.041
Fish gel	δ^15^N	14.45	‰_air N2_	
	δ^13^C	−15.52	‰_VPDB_	
BLS (bovine liver)	δ^15^N	7.65	‰_air N2_	±0.25
	δ^13^C	−21.59	‰_VPDB_	±0.25

*Notes:* IEAE, International Atomic Energy Agency; IRMS, isotope ratio mass spectrometry; VPDB, Vienna Pee Dee Belemnite.

### 2.3 Methods

**FIGURE 2:**
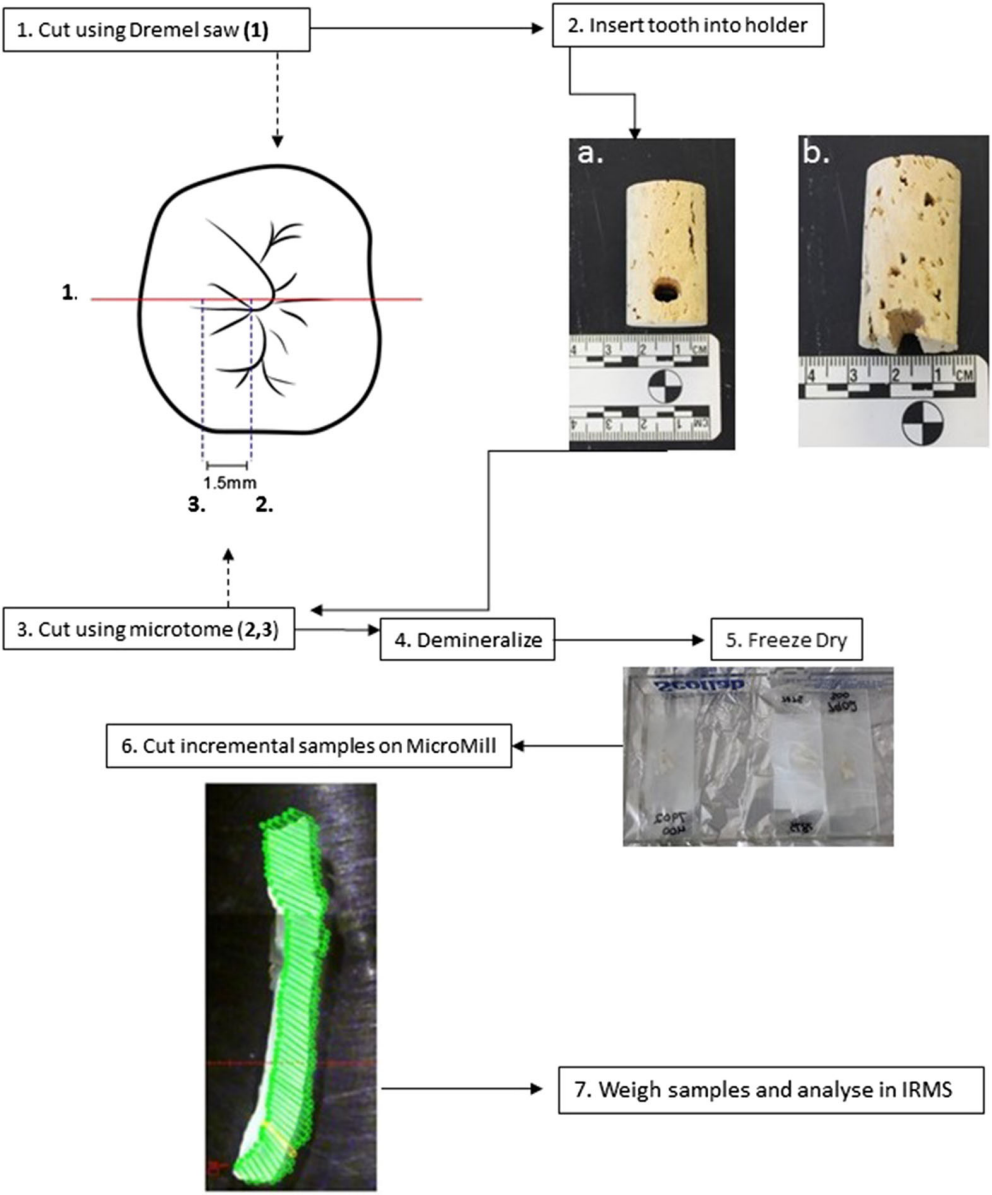
Micro‐sampling workflow. (1) Cut tooth to remove selected root using Dremel saw. (2) Place root in holder (adhesive may be required for b.). (3) Cut lines (2) and (3) using saw microtome. (4) Demineralise dentine sample in 0.5 M HCl. (5) Freeze and freeze dry demineralised collagen, and place it between two glass slides and wrap in Parafilm. (6) Cut incremental samples using Micromill at 0.35 mm. (7) Weigh incremental collagen samples and analyse in isotope ratio mass spectrometer (IRMS) [Color figure can be viewed at wileyonlinelibrary.com]

(see Figure 2 for images and flowchart).

#### 2.3.1 Sample preparation/cutting

Each tooth was sectioned longitudinally, selecting the longest available root and corresponding crown tissue using a Dremel handheld diamond‐edged saw. The roots were secured in a cork holder and placed in a Leica SP1600 Saw Microtome (see workflow chart) used to section the samples because of the precision of the saw. A 1.5 mm thick longitudinal section was cut from the root.

#### 2.3.2 Demineralisation

The longitudinal sections were then demineralised using a modified Longin method.^38^ The sections were demineralised in 0.5 M hydrochloric acid (HCl) at 4°C for approximately 4 days with the acid being changed regularly. When demineralisation was complete, the samples were rinsed 12 times with de‐ionised water to remove contaminants.

#### 2.3.3 Freezing and freeze‐drying

The collagen sample sections were then placed between two glass microscope slides and wrapped in Parafilm® (Bemis Company, Inc., Neenah, WI, USA) to allow the collagen samples to remain flat during the freezing and freeze‐drying process. The demineralised dentine sections were placed in a − 36° freezer overnight. Once frozen, the Parafilm® was punctured, and the samples were lyophilised for 24 h resulting in a solid collagen section. Previous research has shown that the δ^13^C and δ^15^N of dentine samples that have been denatured before lyophilisation are not significantly different to samples from the same incremental section those which have not.^14^ This allows the retention of the microscopically visible incremental structures in the root prior to micro‐sampling.

#### 2.3.4 Incremental sampling

The sections for ET‐7889, ET‐9644, and ET‐7891 were fixed to a glass microscope slide using U.V. Glue (Bondic®). This adhesive was found not to alter the measured δ^13^C and δ^15^N.

Using MicroMill software (New Wave Research) consecutive channels of 1 mm depth, 0.35 mm diameter were cut along the available width of the tooth, following the direction of the Andresen lines (Figure 2 image 6). The milled collagen samples were collected after each channel was cut using a scalpel to pick up the samples off the glass slide and were placed in labelled aluminium containers. The scalpel, drill bit, and surrounding area were cleaned with acetone in between each incremental sample. Each collagen sample was weighed into tin capsules for IRMS analysis. Each incremental sample provided an average weight of 0.34 mg and was analysed once, instead of the standard practise of analysing in duplicate.

#### 2.3.5 Assigning age‐at‐formation to dentine increments

The assessment of the age‐at‐formation was approximated based on the *London Atlas of Tooth Development and Eruption* by AlQahtani et al (2010) and Beaumont and Montgomery (2015).^35^, ^39^ The third molar begins formation between the ages 7 and 9 and reaches apex closure between 17 and 23 years. The approximate formation age was based on the estimated age of apex closure divided by the number of increments, with each increment representing an equal portion of the formation time. In these third molar teeth each increment was calculated to represent approximately 2–4 months.

## 3 RESULTS

The collagen samples of all increments from ET‐7889, ET‐7891, and ET‐9644 had C:N atomic ratio values within the accepted range proposed by Van Klinken (3.1–3.6)^40^ and are reported in Table 2.

**TABLE 2:** Summary statistics for δ^13^C and δ^15^N for all samples

Dentine profile				*δ* ^*13*^ *C*_*VPBD*_ ‰			*δ* ^*15*^ *N*_*AIR*_ ‰		
ID	Tooth	# of increments	Approx. age of apex closure	Min.	Max.	Range	Min.	Max.	Range
ET‐7891	M3	31	17.5 (broken root)	−14.3	−9.4	4.9	9.2	12.4	3.1
ET‐7889	M3	38	23	−18.1	−16.9	3.3	11.3	12.3	1.0
ET‐9644	M3	65	23	−19.1	−18.2	0.9	8.7	10.7	2.0

Because of the low weight achieved the incremental dentine collagen samples acquired through the novel method were analysed once. The incremental samples acquired using Beaumont method 2 were analysed in duplicate by Jackson (2017) and the mean values.

The δ^13^C values of ET‐7889 range from −18.1‰. to −16.9‰. The δ^15^N ratios of ET‐7889 range from 11.3‰ to 12.3‰. The δ^13^C values of ET‐7891 range from −14.3‰ to −9.4‰, with the δ^15^N ratios ranging from 9.2‰ to 12.4‰. The δ^13^C values of ET‐9644 range between −19.1‰ and −18.2‰, with the δ^15^N ratios ranging from 8.7‰ to 10.7‰.

**FIGURE 3:**
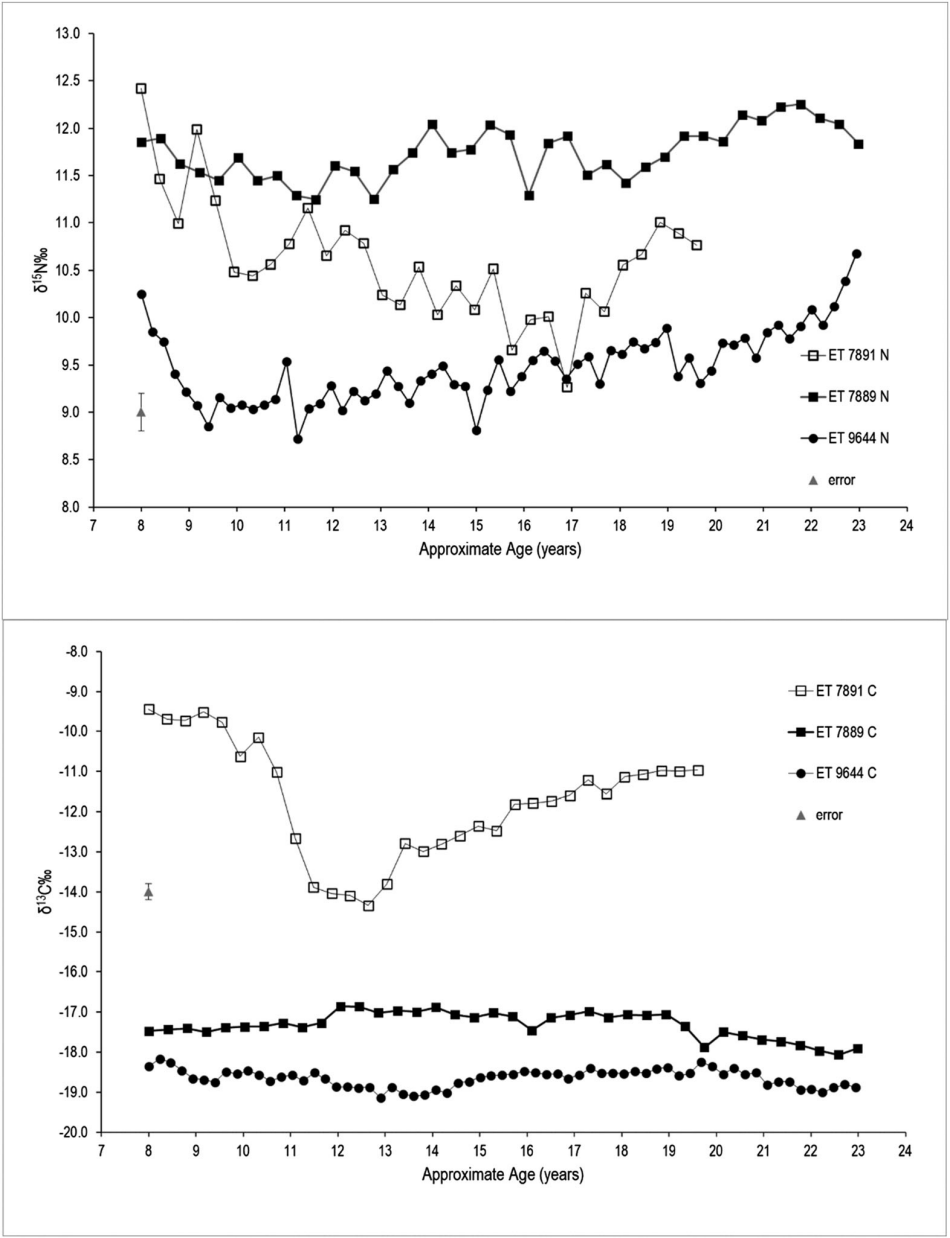
δ^15^N‰ and δ^13^C‰ isotope data from ET 7891, ET 7889, and ET 9644 by 0.35 mm demonstrating dietary differences. Error bar shows ±0.2‰ analytical error

Figure 3 shows the combined δ^13^C and δ^15^N dentine profiles against approximate assigned age for each of the individuals. This demonstrates that there are different dietary inputs and profile patterns between the three individuals.

**FIGURE 4:**
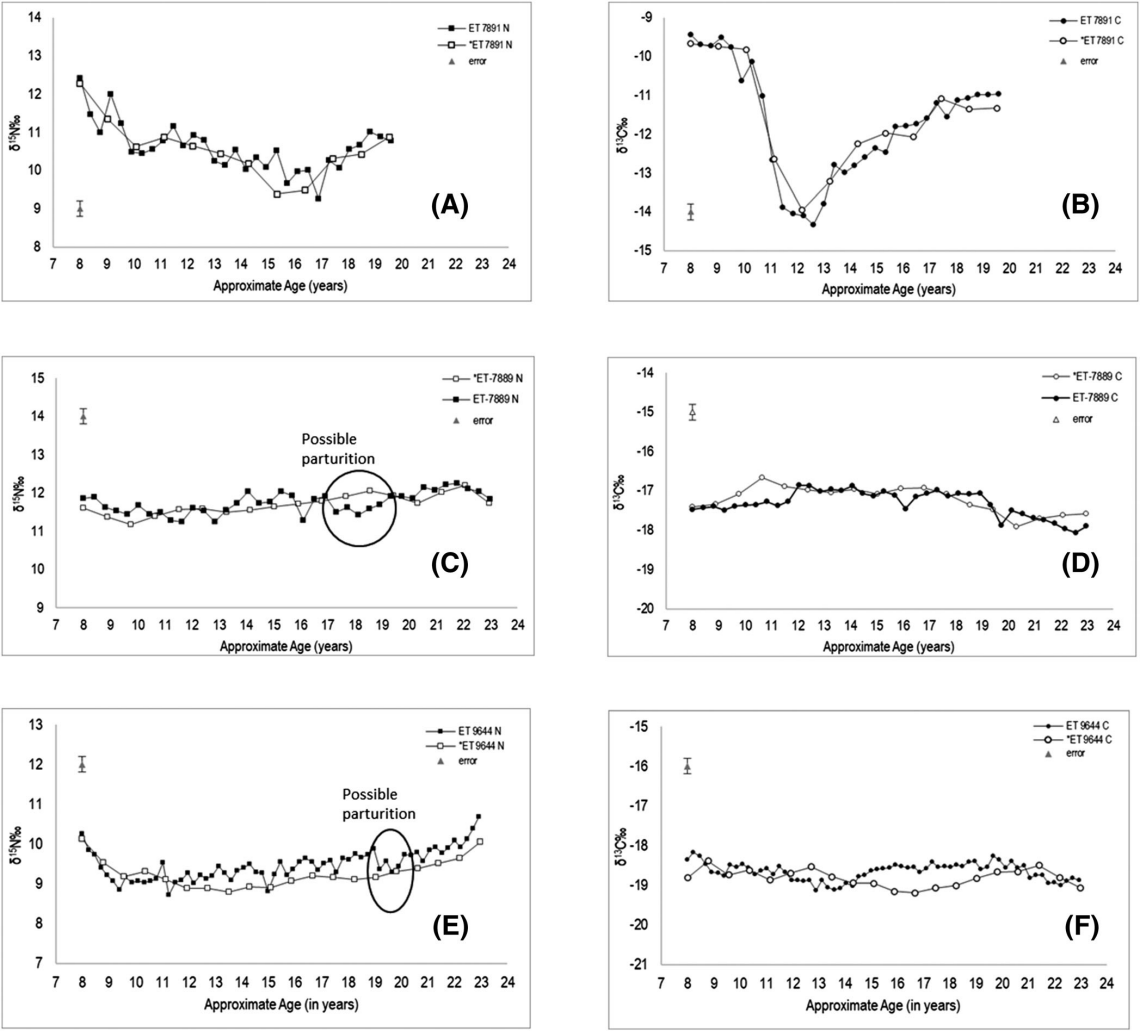
Comparative δ^15^N‰ and δ^13^C‰ isotope data from ET 7891 (A and B), ET 7889 (C and D), and ET 9644 (E and F) for 1 mm sectioning (open circles) (original sample prep and analysis by Jackson^34^) and 0.35 mm sectioning (closed circles). Error bar shows ±0.2‰ analytical error. Possible markers for parturition are shown for ET 7889 and ET 9644

Figure 4 shows the dentine profiles obtained by Beaumont Method 2 with the data from the new micro‐milling method for each individual. This demonstrates that there are a greater number of changes (better temporal resolution) and a higher range of values (less attenuation of the values) in the new data when compared with the 1 mm dentine collagen sections from the same tooth.

## 4 DISCUSSION

Analysis of the three individuals in terms of their dietary history reveals two different patterns (Figure 3). According to the Food and Agriculture Organisation of the United Nations (FAO)^41^ the staple foods in Sudan during the period of tooth growth of these individuals are mainly plant‐based with sorghum, cassava with groundnuts and milk the major sources of protein. Meat and eggs were rarely consumed, and marine foods were most prevalent in southern Sudan and coastal regions. There is evidence for the introduction of maize (a C_4_ plant) as a relief food during famine periods. ET 7889 and ET 9644 have δ^13^C and δ^15^N values which do not vary much, and both are consistent with stable access to a mainly plant‐based diet as described earlier. ET 7889 has slightly higher δ^13^C and δ^15^N throughout the profile than ET 9644 which suggests access to some marine resources: whereas ET 9644 has higher δ^13^C and δ^15^N (probably showing a reduction in trophic level) at the beginning of the profile aged about 8 years but only higher δ^15^N at the end of the profile which suggests some catabolic effect.^42^ ET 7891 has a much more variable profile commencing aged 8 with the highest values of the three individuals for both δ^13^C and δ^15^N, followed by a rapid fall in δ^13^C at the age of 10 years and a corresponding smaller fall in δ^15^N. The δ^13^C in this case is consistent with C_4_ plants forming a major part of the diet but reducing sharply for a period between the ages 10 and 12 when other foods are introduced, before increasing again throughout the rest of the profile and which corresponds with a smaller increase in δ^15^N. All the δ^13^C remains above the highest values for the other two individuals while δ^15^N falls between them. This profile suggests an individual who is consuming C_4_ relief foods with a corresponding catabolic increase in δ^15^N at the start and end of the profile, and a reduction in δ^15^N when access to other foods is available.^42^

The development of the micro‐milling method went through several stages to determine the ideal sample thickness, channel diameter, and order of steps. For example, to drill a sample using the MicroMill, the sample has to be in a solid state: thus the sample would have to be milled either before the dentine collagen was demineralised or after the collagen had been freeze‐dried. During the development of this method, it was determined that there was a large volume of milled sample lost during demineralisation that did not occur if the increments were milled after demineralisation and freeze drying. Cutting the sample channels at an angle, as shown in Figure 2 (6), allows for sampling along the growth lines of the dentine formation. Each section analysed for ET‐7891 and ET‐7889 provides approximately 3 months of isotope ratio data. The incremental sections for ET‐9644 provide approximately two and a half months of isotope ratio data. The 2 to 4 months of isotope data is an improvement on the 6 to 9 months that are provided from these teeth using the incremental methods described in Beaumont et al (2013).^26^ The temporal resolution achievable will vary due to the different growth rates between different tooth types and the length of the root sampled: thus a tooth which takes a long time to develop will have poorer resolution than a fast‐growing tooth, and a tooth with a long root such as the permanent maxillary canine will have better resolution than a short‐rooted third molar.^39^

Figure 4 compares the δ^13^C and δ^15^N profiles achieved using Beaumont Method 2 against this novel method for the same three teeth. Although broadly similar patterns can be seen in the δ^13^C and δ^15^N profiles, additional short‐term changes are visible. Both the novel method and the Beaumont method show general patterns that are similar. The Beaumont method uses between one half to a whole root, depending on size, with 1 mm incremental sections cut horizontally, whereas the novel method uses a 1.5 mm section of root, with 0.35 mm width × 1 mm depth incremental sections cut following the growth pattern of the dentine. Because two of every three 0.35 mm increments co‐formed with the 1 mm increment from the Beaumont method, these result in two points that will average around the 1 mm increment isotopic value. The variation between methods has not significantly altered the values of the data which are obtained from the co‐forming tissues. However, Figure 4 (B and F) show δ^13^C profiles that do not consistently correspond between the data from the 0.35 mm sampling method and the 1 mm sampling method. The lack of corresponding timing of these data points could be related to methodological differences affecting the accuracy of measuring co‐forming tissues. The 1 mm sectioning method relies on cutting increments by hand and by eye rather than using measuring tools and microscopic views. In this pilot study the incremental sections were taken from different roots from the same molar tooth, which may have been different lengths. In a prospective study, the samples for both methods would be taken from the same root. Furthermore, the dentine collagen measurements in this study measured a single (rather than duplicate) collagen sample due to the low average weight (0.35 mg) which could result in outliers or analytical errors being present in the isotopic data set; it is important to ensure that quality parameters such as the C:N ratio are met and any outliers may be detected by observing overall trends in the isotopic profiles.

A further disadvantage of the novel method is the cost of analysis. The novel method produces approximately thrice the number of samples from the Beaumont method, and this is turn can be as many as 20 samples. Each measurement will cost the same as a single bulk sample. Because of this, researchers will need to determine if this increased temporal resolution is necessary to answer the research questions and consider the cost of the method relative to the value of information that can be obtained. The increased temporal resolution allows the measurement of a wider range of values without attenuation of the signals. The δ^13^C and δ^15^N from this and previous methodological studies demonstrate how the averaging caused by larger samples can mask changes in the isotope values.

Jackson (2017) used the Beaumont method to investigate the possibility of identifying the effects of pregnancy and parturition on the δ^15^N values from dentine collagen.^34^ Given that these teeth were collected as part of a group where parturition was known to have occurred during the age the teeth were forming (although detailed medical histories are not available), the novel method described in this paper has identified periods in the isotope profiles of ET‐7889 and ET‐9644 where potential pregnancy and parturition may be observed. The period identified as potential pregnancy in the isotopic profile of ET‐7889 occurred between 17 and 19 years (Figure 4C) with a decrease in δ^15^N values of ≥0.6‰ while the δ^13^C values remained relatively steady. The period identified in the isotopic profile for ET‐9644 occurred between 19 and 20 years (Figure 4E) with a similar 0.6‰ decrease in δ^15^N values. Providing an exact isotopic profile which could be applied to other samples to determine pregnancy in an individual is not possible with the limited number of individuals analysed; however, ET 7889 and ET 9644 show a pattern of isotope variation similar to that which has been observed by D'Ortenzio et al (2015)^43^ who analysed hair keratin to research the effects of physiological stress on δ^13^C and δ^15^N values. The observed pattern in the isotopic values from incremental sampling of hair grown by two women during pregnancies showed a decrease in δ^15^N values that persists across multiple incremental data points. Although hair keratin and dentine collagen have different chemical makeups, the similarities between the isotopic profiles indicate that the increased temporal resolution in the dentine collagen may be capable of providing information that was not previously observable. The observed isotopic pattern was not seen in the less detailed isotopic data from the Beaumont method.

The level of collagen preservation will be a vital aspect for the use of this novel method on archaeological dentine samples.^14^, ^44^ The preservation of samples was not a concern with these modern samples, and further work is being carried out to repeat the comparison between the two methods using well‐preserved archaeological teeth. An increased temporal resolution applied to deciduous teeth could also provide more detailed information about maternal health, pregnancy, and weaning practices.

## 5 CONCLUSIONS

The isotopic data generated using longitudinal sectioning and micro‐milling in the direction of the incremental structures matches well with previous data produced with Beaumont Method 2 from the same tooth while demonstrating that there is more detailed variation which has been lost in the established method.

The study demonstrates the potential for a method of precision sampling of human dentine collagen along incremental structures which can improve the temporal resolution currently possible with other micro‐sampling approaches and thus the ability to investigate shorter‐term periods of nutritional or physiological stress, including potential seasonal dietary changes. Further work is in progress to determine the effect of taphonomic and diagenetic modifications and preservation of collagen on the reliability of the method.

6

### PEER REVIEW

The peer review history for this article is available at https://publons.com/publon/10.1002/rcm.9305.

## Data Availability

Data available in article supplementary material.
